# Treatment Initiation, Program Attrition and Patient Treatment Outcomes Associated with Scale-Up and Decentralization of HIV Care in Rural Malawi

**DOI:** 10.1371/journal.pone.0038044

**Published:** 2012-10-15

**Authors:** Megan McGuire, Loretxu Pinoges, Rupa Kanapathipillai, Tamika Munyenyembe, Martha Huckabee, Simon Makombe, Elisabeth Szumilin, Annette Heinzelmann, Mar Pujades-Rodríguez

**Affiliations:** 1 Epicentre, Clinical Research Department, Nairobi, Kenya; 2 Epicentre, Clinical Research Department, Paris, France; 3 Médecins Sans Frontières, Chiradzulu, Malawi; 4 Monash Medical Centre, Melbourne, Australia; 5 Clinical HIV Unit, Ministry of Health, Lilongwe, Malawi; 6 Médecins Sans Frontières, Paris, France; University of Cape Town, South Africa

## Abstract

**Objective:**

To describe patient antiretroviral therapy (cART) outcomes associated with intensive decentralization of services in a rural HIV program in Malawi.

**Methods:**

Longitudinal analysis of data from HIV-infected patients starting cART between August 2001 and December 2008 and of a cross-sectional immunovirological assessment conducted 12 (±2) months after therapy start. One-year mortality, lost to follow-up, and attrition (deaths and lost to follow-up) rates were estimated with exact Poisson 95% confidence intervals (CI) by type of care delivery and year of initiation. Association of virological suppression (<50 copies/mL) and immunological success (CD4 gain ≥100 cells/µL), with type of care was investigated using multiple logistic regression.

**Results:**

During the study period, 4322 cART patients received centralized care and 11,090 decentralized care. At therapy start, patients treated in decentralized health facilities had higher median CD4 count levels (167 vs. 130 cell/µL, *P*<0.0001) than other patients. Two years after cART start, program attrition was lower in decentralized than centralized facilities (9.9 per 100 person-years, 95% CI: 9.5–10.4 vs. 20.8 per 100 person-years, 95% CI: 19.7–22.0). One year after treatment start, differences in immunological success (adjusted OR = 1.23, 95% CI: 0.83–1.83), and viral suppression (adjusted OR = 0.80, 95% CI: 0.56–1.14) between patients followed at centralized and decentralized facilities were not statistically significant.

**Conclusions:**

In rural Malawi, 1- and 2-year program attrition was lower in decentralized than in centralized health facilities and no statistically significant differences in one-year immunovirological outcomes were observed between the two health care levels. Longer follow-up is needed to confirm these results.

## Introduction

In sub-Saharan Africa, approximately 5,200 people became newly infected with HIV each day in 2008, and 3835 died daily from AIDS-related conditions [1]. Malawi bears one of the largest HIV-related health burdens in the world. An estimated 1 million people are living with HIV/AIDS, and approximately 84,000 new infections and 68,000 HIV-related deaths occur annually [2]–[4]. The Malawian Ministry of Health adopted an ambitious public health approach for combined antiretroviral therapy (cART) scale-up in 2004 [2], [5], [6]. At the end of 2008, it was estimated that 56% of people in urgent need for ART were receiving treatment [7], [8] and a total of 147,479 individuals were alive and receiving therapy in the country [9]. Over 80% of the Malawian population lives in rural settings [10], but most patients access treatment through major urban sites or district hospitals.

The effectiveness of cART in terms of mortality reduction at both population [11] and individual level [12], [13] has been well documented. However, important challenges limit scaling-up of care, including difficult access of rural populations to centralized care, and shortage of health workers. With only one doctor per 100,000 people [14], [15], health care in Malawi is generally provided by clinical officers and, as the HIV case load increases, delegation of tasks to medical assistants and nurses is implemented.

In the Chiradzulu district, located in Southern Malawi, 1 in 4 adults are estimated to be living with HIV infection. In 2003 decentralized HIV care started being provided by mobile health teams every 2 weeks in 10 peripheral health facilities [16]. By mid-2004 one quarter of all cART initiations in the district occurred in decentralized health facilities. The size of the mobile teams and the number of days when HIV care services were provided increased over time. In 2007 nurses working at the health centers were trained to initiate cART and to monitor the clinical evolution of HIV-infected patients, further accelerating the scale-up of HIV care provision. The feasibility and safety of wide scale-up of HIV care through decentralization to primary health centers, and task-shifting to lower-level medical cadres has been highly debated [12], [17]–[21]. Results from effectiveness evaluations are inconsistent [22], [23].

Here we describe temporal trends in patient characteristics at cART start and compare outcomes in HIV-infected patients receiving decentralized versus centralized care in Chiradzulu, Malawi.

## Materials and Methods

### Study setting and participants

The Chiradzulu HIV program was established in 1996 as a collaboration between the Ministry of Health of Malawi and Médecins Sans Frontières (MSF). Free provision of cART at the district hospital started in August 2001 [24], [25]. To increase access to HIV care in this large and populated district (>310,000 people) with an estimated HIV prevalence of 20% in 2007, decentralization of care to 10 peripheral health facilities started in 2003 using mobile medical teams. Health service decentralization included cART initiation and clinical follow-up of patients by clinical officers. As of May 2007, nurses and medical assistants began initiating therapy to adults. During follow-up, patients could access HIV care in any of the health facilities of the district. Lay community workers carried out HIV testing, daily registration and scheduling of patients and routinely traced defaulters within 2 days of missing their clinic appointments. Information about patient death was collected through several mechanisms including reports from family members at the health facilities, tracing by lay community workers and verification of inpatient records. All services related to HIV care, including laboratory testing, provision of cART, management of opportunistic infections and hospitalization, were free.

Eligibility criteria for cART initiation followed World Health Organization (WHO) treatment guidelines: CD4 cell count <200 cells/µL (<250 since January 2007), clinical stage 4 irrespective of CD4 cell count, and since January 2007, clinical stage 3 irrespective of CD4 cell count [25]. After cART start, and once the patient's health stabilized, clinical visits were scheduled at a maximum of every 3 months. Group and individual counseling, including adherence support, was provided by peer workers and trained counselors. Routine CD4 count testing was performed before and every 12 months after starting cART. Viral load testing was only done when treatment failure was suspected.

### Study design and data collection

First, a retrospective cohort analysis of monitoring data from patients starting cART between August 2001 and December 2008 at the district hospital and at 10 rural health centers was conducted. Sociodemographic, clinical, and treatment information were routinely recorded on the patient files. Carbon-copied duplicate forms were then sent to the hospital for data entry in an electronic database (FUCHIA; Epicentre, Paris, France) and record filing. Standard data consistency checks (e.g. consistency between key dates such as dates of enrolment, cART start or date of death) and verifications (e.g. regular cross-verification of paper forms and laboratory information against the information entered in the database) were routinely done on and off site.

Second, a cross-sectional survey was carried out in February–July 2009 to determine immunovirological outcomes among patients receiving cART for 12±2 months. A random selection of 702 eligible patients was invited to participate in the evaluation (312 in centralized and 390 in decentralized care). The sample size was estimated to detect a 10% difference in virological failure between patients receiving centralized versus decentralized care, assuming a significance level of 5% and 80% power. Fifty-two (7.4%) women were excluded due to current pregnancy or breastfeeding, 9 (1.3%) had died, 3 (0.4%) had been transferred outside the program, 13 (1.8%) were lost to follow-up and 7 (1.0%) declined participation. Table S1 compares the characteristics of patients who were included and excluded from the study. A total of 618 patients were enrolled and participated in the survey.

An independent clinical team scheduled appointments for the study patients, obtained informed consent, and conducted the cross-sectional evaluation. Physical examination by clinicians, including assessment of cART adherence during the previous month using a 10-point visual analogue scale, and blood draws for laboratory testing, were performed at the facilities. Results were recorded on standardized questionnaires. Data were double-entered into a Microsoft Access database.

Blood specimens were collected and labeled. CD4 cell counts were quantified using semi-automated machines (Cyflow counter, Partec, Münster, Germany) at the laboratory of the Chiradzulu district hospital. Plasma samples were stored at -20°C and transported to the DREAM Laboratory (Blantyre, Malawi) weekly for viral load quantification with the VERSANT HIV-1 RNA bDNA assay (version 3.0, Siemens Healthcare Diagnostics, Deerfield, IL, USA; lower limit of detection was 50 copies/mL).

### Statistical analysis

#### Retrospective cohort analysis

We used individual patient monitoring data to compare patient characteristics at cART start by calendar period of cART initiation (2001–2002, 2003–2004, 2005–2006, 2007–2008) and type of care delivery (centralized or decentralized). Type of care was defined as patient attendance to the district hospital or to health centers, respectively, for at least 90% of all consultations since cART initiation. Characteristics between the two care groups were compared using chi-squared tests or Fisher exact tests for categorical and Wilcoxon rank sum tests for continuous variables.

Time of follow-up for each patient started at the time of cART initiation and was censored at the earliest of the following dates: death, transfer outside the program, last clinic visit or 2 years. One-year mortality, lost to follow-up, and attrition (deaths and lost to follow-up) rates were estimated with exact Poisson 95% confidence intervals (CI). Lost to follow-up was defined as missing a scheduled appointment by 2 months or more.

#### Cross-sectional survey

The proportions of patients included in the cross-sectional evaluation with undetectable viral load, virological failure (>5000 copies/mL), and CD4 cell counts <200 cells/µL, and gains in CD4 counts since the start of therapy were stratified according to the type of care received. In sensitivity analyses, the rate of virological failure was estimated assuming both, that patients who were alive and in care 12 months after cART start but who did not participate in the cross-sectional study had experienced the same rate of virological failure as patients who participated in the evaluation; and that all patients who died or were lost to follow-up between 6 and 12 months after cART start had experienced virological failure. Patients who died or were lost to follow-up within 6 months of therapy initiation were excluded from this estimation because in WHO guidelines only patients who receive cART for more than 6 months are considered in the definition of treatment failure. Multiple logistic regression was used to compare virological suppression (<50 copies/mL) and immunological success (CD4 gain since treatment start ≥100 cells/µL) at 12 months of cART among patients aged 2 years or more, and 5 years or more, respectively. Factors considered in multivariable models were residence in the Chiradzulu district (yes/no), sex, age in quartiles (≤25, 26–35, 36–42, and ≥43 years), body mass index (<18.5 and ≥18.5 kg/m^2^ for patients aged >18 year old, and equivalent age and sex specific BMI cut-off points for the 2–18 year old group [26]), highest education level achieved (none, primary, and high school or more), and adherence in last 4 weeks (<90%, 90% and 100%) at the time of the survey; and record of history of cART use, CD4 cell count (≤100, >100 cells/µL, and missing) and clinical stage (1 or 2; and 3 or 4) at cART start. The initial CD4 cell count was defined as the closest recorded measurement to therapy start between 3 months before and 1 month after this date.

All factors associated with a CD4 gain ≥100 cells/µL (or with virological suppression) in univariable analyses (*P*<0.20) were included in the corresponding multivariable models. Likelihood ratio tests for associations across categories of variables were calculated. Sensitivity analyses excluding observations with missing CD4 cell count and clinical stage data (n = 60) were also performed. All data analyses were carried out using STATA statistical software, version 11.2 (StataCorp, College Station, TX, USA). Statistical significance was defined by two-sided *P* values of <0.05.

### Ethics

The study was approved by the National Health Science Research Committee of Malawi. Written patient informed consent was obtained from participants or from guardians of participants for minors before participation in the cross-sectional survey.

## Results

### Patient characteristics by type of care delivery

**Figure 1:**
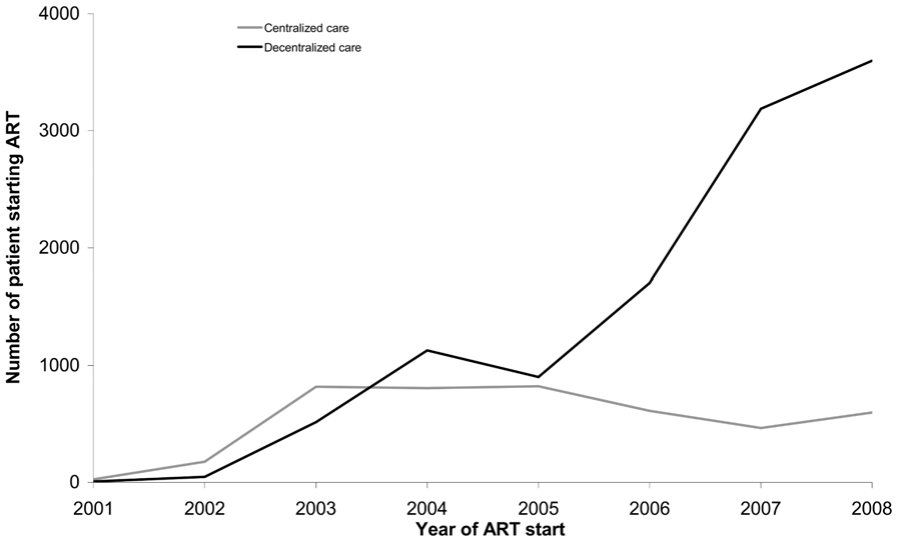
Trends in cART enrollment in centralized and decentralized sites, Chiradzulu, Malawi, 2001–2008.

A total of 17,699 patients started cART between August 1, 2001 and December 31, 2008. Of these, 4322 received centralized care, and 11,090 decentralized care. The other 2287 patients who received mixed care were excluded from the analysis. As scaling-up through decentralization to peripheral health facilities progressively evolved in the program, the number of cART initiations increased, with a steep raise observed during the 2007–2008 period (Figure 1). During the period 2001–2002, 78.5% of initiations took place at centralized level. By the end of 2008, 86.5% of cART initiations occurred in health centers. The number of therapy initiations in decentralized facilities greatly increased over time, from 56 in 2001–2002 to 3,598 in 2008. In contrast, the number of patients who were started on cART at hospital level, increased until the end of 2004, and then started declining slowly over time.

In each time period, higher proportions of men were initiated on cART in centralized than in decentralized facilities (*P*<0.001, 2003–2008), and median age was similar in patients who received centralized or decentralized care (Table 1). As the program scaled up and cART became more accessible, an increasing number of patients started cART with less advanced HIV disease. The initial median CD4 cell count increased from 122 cells/µL in 2001–2002 to 149 in 2007–2008 at centralized level, and from 93 cells/µL in 2001–2002 to 176 in 2007–2008 at decentralized level. Median CD4 cell count increases were higher in patients receiving decentralized care than in those treated in centralized health facilities (*P*<0.001 after 2002).

**Table 1:** Patient characteristics at cART start by type of care delivery, Chiradzulu, Malawi, 2001–2008.

	2001–2002			2003–2004			2005–2006			2007–2008		
	Centralized	Decentralized	*P*	Centralized	Decentralized	*P*	Centralized	Decentralized	*P*	Centralized	Decentralized	*P*
Patients ART initiated, n	204	56		1622	1643		1434	2605		1062	6786	
**Sex**, n (%)												
Men	86 (42.2)	20 (35.7)	0.39	615 (37.9)	488 (29.7)	<0.001	615 (42.9)	769 (29.5)	<0.001	461 (43.4)	2436 (35.9)	<0.001
Women	118 (57.8)	36 (64.3)		1007 (62.1)	1155 (70.3)		819 (57.1)	1836 (70.5)		601 (56.6)	4350 (64.1)	
**Age group, n (%)**												
≤25 yr	31 (15.4)	11 (19.6)	0.48	276 (17.6)	334 (20.7)	0.054	227 (16.1)	504 (19.5)	0.001	165 (15.6)	1314 (19.4)	0.002
26–35 yr	99 (48.2)	21 (37.5)		580 (36.9)	577 (35.7)		566 (40.1)	959 (37.0)		400 (37.8)	2518 (37.2))	
36–42 yr	40 (19.9)	14 (25.0)		371 (23.6)	335 (20.7)		325 (23.0)	508 (19.6)		245 (23.2)	1290 (19.1)	
≥43 yr	31 (15.4)	10 (17.9)		344 (21.9)	369 (22.8)		294 (20.8)	620 (23.9)		248 (23.4)	1644 (24.3))	
Unknown	3	0		51	28		22	14		4	20	
**Age , years**												
Men, median [IQR]	35.8 [32.0–42.1]	36.3 [31.0–40.5]	0.49	37.1 [30.6–44.0]	37.5 [30.7–44.1]	0.84	36.9 [30.2–43.8]	36.4 [29.9–45.2]	0.55	36.9 [31.2–44.3]	36.5 [30.2–45.1]	0.90
Women, median [IQR]	31.0 [26.8–36.1]	32.8 [28.0–39.9]	0.16	33.1 [27.6–40.1]	33.2 [26.9–41.1]	0.65	32.6 [27.8–40.1]	33.1 [27.1–40.8]	0.26	33.1 [27.9–41.1]	32.8 [27.0–40.5]	0.23
**History of cART use**, n (%)												
Yes	2 (1.0)	0	-	76 (4.7)	12 (0.7)	<0.001	257 (17.9)	327 (12.5)	<0.001	86 (8.1)	322 (4.7)	<0.001
**CD4 cell count**, cells/µL^a^												
Tested, n (%)	166	42		864	771		428	1253		864	5565	
Median, [IQR]	122 [50–180]	93 [59–145]	0.24	119 [59–186]	145 [88–200]	<0.001	122 [66–193]	144 [82–207]	0.001	149 [74–219]	176 [105–229]	<0.001
**Clinical stage**, n (%)												
1 or 2	41 (20.8)	9 (17.3)	0.57	212 (14.5)	279 (18.3)	0.005	178 (16.6)	587 (26.5)	<0.001	317 (30.6)	3554 (53.9)	<0.001
3 or 4	156 (79.2)	43 (82.7)		1255 (85.5)	1247 (81.7)		895 (83.4)	1626 (73.5)		719 (69.4)	3035 (46.1)	
Unknown	7	4		155	117		361	392		26	197	
**Body mass index**, n (%)^b^												
<18.5 kg/m^2^	54 (32.9)	14 (25.0)	0.27	376 (28.3)	551 (38.4)	<0.001	349 (28.1)	908 (36.6)	<0.001	238 (32.8)	2070 (31.6)	<0.001
≥18.5 kg/m^2^	110 (67.1)	42 (75.0)		955 (71.7)	883 (61.6)		892 (71.9)	1570 (63.4)		762 (76.2)	4472 (68.4)	
Unknown	35	0		232	172		149	80		29	19	
**cART regimen**, n (%)												
3TC d4T NVP	61 (29.9)	21 (37.5)	0.49	1563 (96.4)	1583 (96.3)	0.96	1306 (91.1)	2468 (94.7)	<0.001	854 (80.4)	6207 (91.5)	<0.001
3TC AZT NVP	133 (65.2)	32 (57.1)		46 (2.8)	48 (2.9)		34 (2.4)	56 (2.1)		26 (2.4)	317 (4.7)	
Other	10 (4.9)	3 (5.4)		13 (0.8)	12 (0.7)		94 (6.6)	81 (3.1)		182 (17.1)	262 (3.9)	

a >5 years (n = 14,520),

b >2 years (n = 14,962).

Note: 3TC, lamivudine; cART, combined antiretroviral therapy; AZT, zidovudine; d4T, stavudine; IQR, interquartile range; NVP, nevirapine; yr, years.

Similarly, the proportion of patients in advanced clinical stage decreased over the study period from 79.2% in 2001–2002 to 69.4% in 2007–2008 at centralized level, and from 82.7% in 2001–2002 to 46.1% in 2007–2008 at decentralized level. At cART start and after the year 2002, the proportions of patients with clinical stage 1 or 2 and with high median CD4 cell counts were higher in patients treated in decentralized facilities than in those who received centralized care.

### Mortality and program attrition by type of care delivery

**Figure 2:**
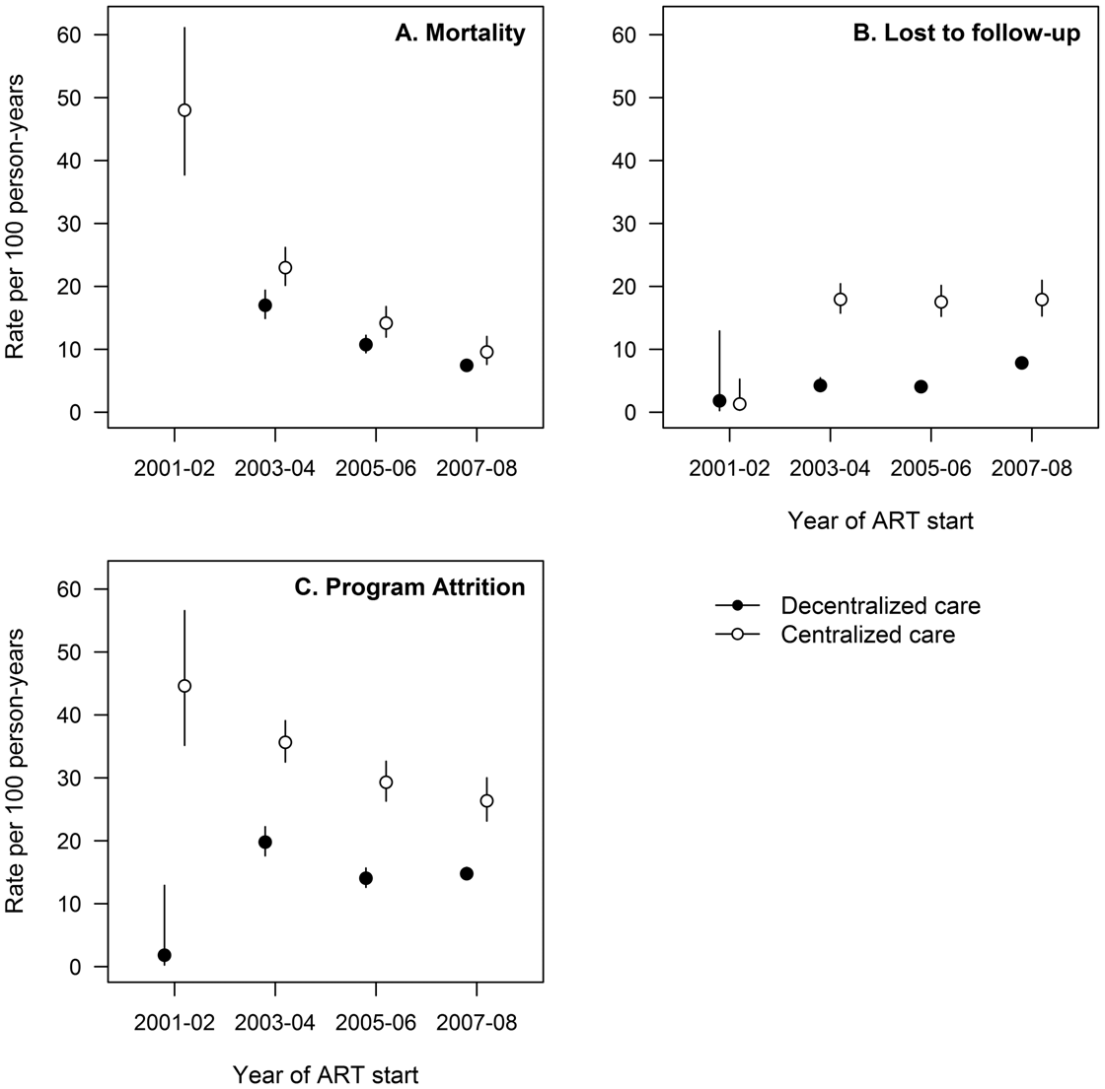
Evolution of mortality, lost to follow-up, and attrition 1 year after cART start.

Overall rates of mortality and program attrition among patients who initiated therapy were 11.5 per 100 person-years (95% CI: 10.9–12.1) and 19.5 per 100 person-years (95% CI: 18.5–20.2) at 1 year of cART start and 6.9 per 100 person-years (95% CI: 6.6–7.3) and 12.6 per 100 person-years (95% CI: 12.2–13.1) at 2 years, respectively. As the program scaled up, patient attrition and mortality decreased in both centralized and decentralized health facilities (Figure 2).

**Figure 3:**
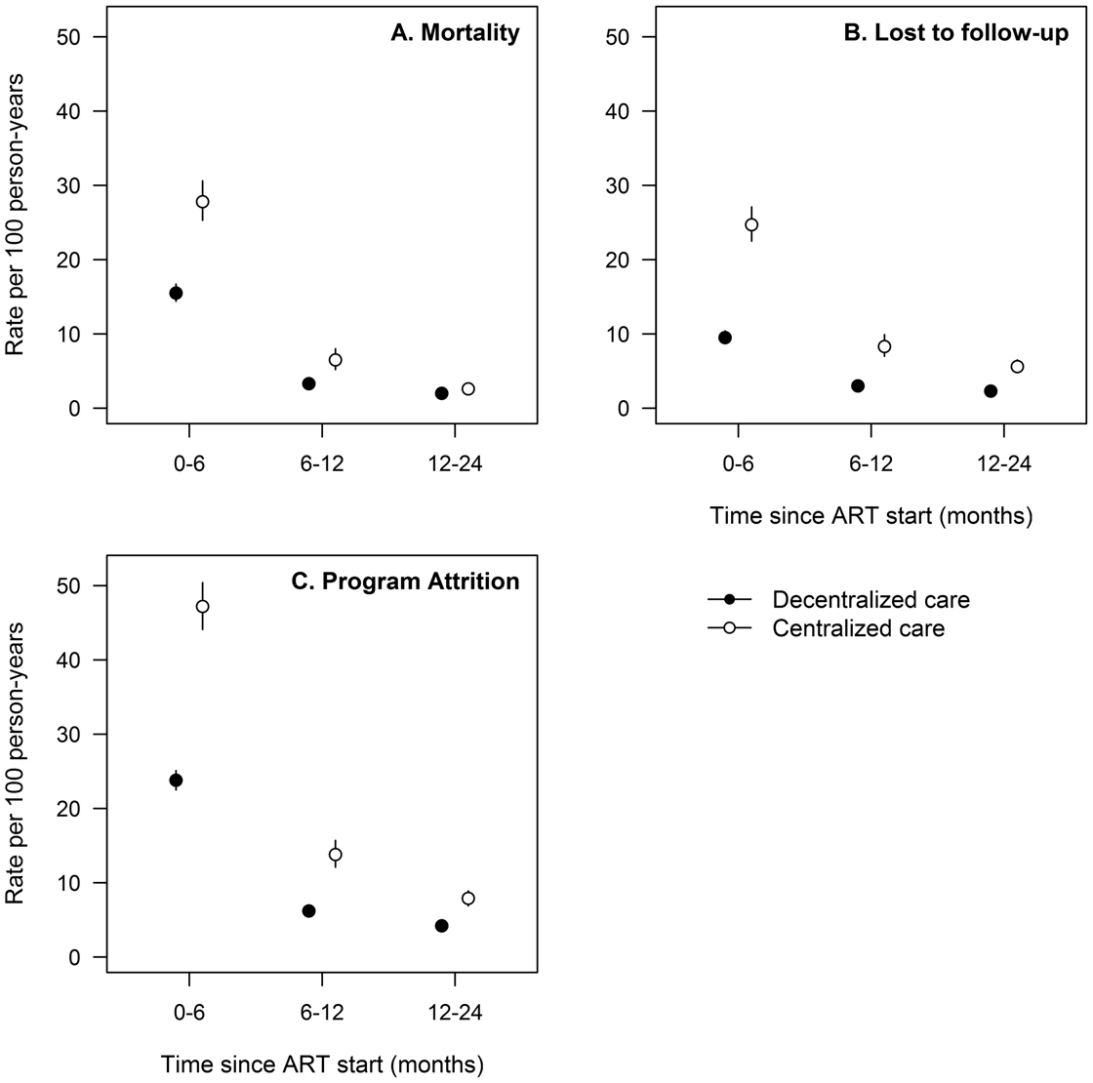
Evolution of rates of mortality, lost to follow-up, and attrition after cART initiation.

During the first year of cART use, mortality and attrition rates were higher in patients treated at central level than in those treated in decentralized health centers (Figure 3). At 6 months of cART use, rates of mortality were 27.8 per 100 person-years (95% CI: 25.3–30.6) at centralized level, and 15.5 per 100 person-years (95% CI: 14.4–16.7) at decentralized level. In the 6–12 month period of cART use, mortality rates were 6.5 per 100 person-years (95% CI: 5.2–8.0) at centralized level, and 3.3 per 100 person-years (95% CI: 2.9–3.9) at decentralized level.

Six-month rates of attrition were 47.2 per 100 person-years (95% CI: 44.1–50.4) at central level, and 23.8 per 100 person-years (95% CI: 22.5–25.1) at decentralized level; and 6–12 month attrition rates were 13.8 per 100 person-years (95% CI: 12.1–15.7) at central level, and 6.2 per 100 person-years (95% CI: 5.5–6.9) at decentralized level.

### One-year immunovirological outcomes and associated risk factors

Six hundred and eighteen patients had immunovirological data at 1 year of cART. Of them, 279 (45.2%) received centralized care and 339 (54.8%) decentralized care.

Of the 584 patients older than 5 years old, 10 (3.8%) patients who received centralized care and 3 (0.9%) treated in health centers had CD4 counts <100 cells/µL (*P* = 0.02). Median CD4 cell gain in the same age group was higher in individuals followed at decentralized health facilities than in those receiving centralized care (+167 cells/µL vs. +145; *P* = 0.03). After 1 year of therapy, 66.9% of patients had CD4 gains of 100 cells/µL or greater, 70.2% of decentralized and 62.7% of centralized patients. In contrast, 57.8% of patients had undetectable viral load one year after ART initiation, 56.6% of decentralized and 59.1% of centralized patients. Only 35 (5.7%) patients had viral load >5000 copies/mL. In sensitivity analysis we estimated an overall failure rate in the program of 9.4% (95% CI: 8.9–9.9%).

Although patients followed at decentralized level, were less likely to achieve virological suppression after 1 year of therapy (adjusted OR [aOR] = 0.80, 95% CI: 0.56–1.14) and more likely to achieve CD4 gains ≥100 cells/µL (aOR = 1.23, 95% CI: 0.83–1.83), these associations were not statistically significant (*P* = 0.21, and 0.30, respectively). Women (aOR = 1.59, 95% CI: 1.08–2.34; *P* = 0.02), and patients with no history of ART use at therapy initiation (aOR = 2.99, 95% CI: 1.13–7.88; *P* = 0.02) were more likely to achieve CD4 gains ≥100 cells/µL after 1 year of cART (Table 2). Older patients were more likely to be virologically suppressed after cART start (aOR = 1.84, 95% CI: 1.03–3.29 for the 36–42 year group; and aOR = 2.15, 95% CI: 1.22–3.81 for the ≥43 year group, compared to patients aged ≤25 years). Sensitivity analyses restricted to patients with complete CD4 count data showed similar results (data not shown).

**Table 2:** Factors associated with immunovirological outcomes 1 year after cART start, Chiradzulu, Malawi.

		CD4 gain ≥100 cells/µL^a^ (N = 550)					Virological suppression (N = 605)				
		No. of patients	Unadjusted OR (95% CI)	*P*	Adjusted OR^c^ (95% CI)	*P*	No. of patients	Unadjusted OR (95% CI)	*P*	Adjusted OR^f^ d (95% CI)	*P*
***Characteristics at cross-sectional evaluation***											
**Type of care**	Centralized	241	1	0.06	1	0.30	279	1	0.53	1	0.21
	Decentralized	309	1.41 (0.98–2.01)		1.23 (0.83–1.83)		339	0.90 (0.65–1.24)		0.80 (0.56–1.14)	
**Residing in the district**	No	161	1	0.18	1	0.76	180	1	0.11	1	0.21
	Yes	389	1.30 (0.88–1.91)		1.07 (0.70–1.62)		438	1.33 (0.94–1.89)		1.27 (0.87–1.85)	
**Highest education level**	No education	149	1	0.06	1	0.35	192	1	0.18	1	0.29
	Primary school	219	0.63 (0.40–1.00)		0.71 (0.44–1.13)		237	1.38 (0.93–2.03)		1.28 (0.86–1.92)	
	High school or more	182	0.59 (0.36–0.95)		0.78 (0.46–1.34)		189	1.01 (0.68–1.52)		0.95 (0.60–1.51)	
**Sex**	Male	199	1	0.001	1	0.02	232	1	0.13	1	0.22
	Female	351	1.82 (1.26–2.62)		1.59 (1.08–2.34)		386	1.29 (0.93–1.79)		1.24 (0.88–1.76)	
**Age group**	≤25 yr	39	1	0.05	1	0.15	87	1	0.06	1	0.06
	26–35 yr	219	1.06 (0.50–2.27)		1.13 (0.52–2.45)		223	1.55 (0.94–2.56)		1.69 (0.98–2.91)	
	36–42 yr	142	0.66 (0.30–1.43)		0.79 (0.36–1.76)		149	1.74 (1.02–2.97)		1.84 (1.03–3.29)	
	43+ yr	150	0.61 (0.28–1.31)		0.67 (0.31–1.48)		159	2.05 (1.20–3.48)		2.15 (1.22–3.81)	
**Body mass index, kg/m^2^** b	<18.5	68	1	0.67			75	1	0.16	1	0.16
	≥18.5	480	1.12 (0.66–1.91)				530	0.70 (0.42–1.16)		0.69 (0.41–1.17)	
**Adherence in last 4 weeks**	<90%	54	1	0.66			60	1	0.80		
	≥90%	195	1.12 (0.59–2.14)				217	0.86 (0.48–1.55)			
	100%	300	0.94 (0.51–1.74)				340	0.82 (0.47–1.45)			
**CD4 cell count, cells/µL** a	<200	110	NA				115	1	0.44		
	≥200	440	NA				469	0.85 (0.56–1.29)			
**HIV-1 RNA, copies/mL**	Detectable (≥50)	224	1	0.37			261	NA			
	Undetectable (<50)	326	1.18 (0.82–1.69)				357	NA			
***Clinico-immunological factors at cART start***											
**History of cART use**	Yes	20	1	0.003	1	0.02	28	1	0.40		
	No	530	3.97 (1.55–10.12)		2.99 (1.13–7.88)		590	1.39 (0.65–2.96)			
**Clinical stage**	3 or 4	263	1	0.74			296	1	0.72		
	1 or 2	273	1.02 (0.71–1.47)				306	0.87 (0.63–1.21)			
	Unknown	14	0.66 (0.22–1.96)				16	0.88 (0.32–2.42)			
**CD4 cell count, cells/µL** a	≤100	131	1	0.47			131	1	0.64		
	>100	419	0.86 (0.56–1.31)				419	0.87 (0.58–1.30)			
	Unknown	0					30	0.70 (0.32–1.57)			

a Exclusion of patient aged <5 years (n = 38);

b Exclusion of patients of <2 years (n = 11);

c OR adjusted for type of care, district of residence, highest education level, sex, age group and history of cART use;

d OR adjusted for type of care, district of residence, highest education level, sex, age group and body mass index.

Note: cART, combined antiretroviral use, NA, non applicable; OR, odds ratio; yr, years.

## Discussion

Expanding access to cART is essential to reduce AIDS-related mortality [27]. This study describes early therapy outcomes among patients treated in a large rural HIV program where extensive scale-up of care was achieved through both delegation of clinical work to non-physician medical personnel and decentralization of health services. Our findings suggest that decentralization of care to primary health facilities is not only associated with increased access to cART in terms of numbers of patients receiving therapy, but also with earlier treatment start in the course of the disease and lower program attrition. Furthermore, in this setting, we did not observe statistical significant differences in immunological and virological outcomes between patients who received decentralized or centralized care after 1 year of cART.

In the Chiradzulu program access to HIV care improved over time, as shown by the increase in the number of patients starting cART and by both, the proportion of patients started on cART at early stages of HIV disease (e.g. WHO clinical stage 1 or 2) and patient median CD4 cell counts at central and decentralized levels. Compared to patients who received decentralized care, those treated at central level were more likely to be men and to be severely immunosuppressed, but less likely to have low BMI at therapy initiation. The higher median CD4 cell count observed in patients who received centralized care could be explained by a combination of the existence of in-patient care and a later presentation of male patients compared to women [28].

Overall program attrition (combined mortality and lost to follow-up) decreased over time and, despite the increasing number of patients started and followed on therapy in decentralized health facilities, this decrease was more important at decentralized care level. After 1 year of cART, 83% of patients continued receiving HIV care (79% after 2 years). These findings are comparable or better than those reported in other African HIV programs, including other MSF programs, where patients were primarily treated in hospitals or urban locations [12], [29]–[32]. They are also similar to weighted mean rates estimated in a systematic review of patient retention in sub-Saharan African HIV programs [33]. The low attrition rate observed among decentralized patients in Chiradzulu could be related to the reduction of patient travel time and costs achieved through decentralization, both factors identified as barriers to HIV care in previous studies [34], [35], and/or to the higher proportion of patients who were living outside the Chiradzulu district at central level.

Virological suppression 1 year after therapy start was observed in 58% of patients. Although this figure is lower than the estimated median proportion of patients virologically suppressed reported in a recent systematic review including data from 29 HIV programs in sub-Saharan Africa, it falls within the range of published estimates (median across studies was 82%, range 51%–97%) [2]. While most studies conducted in sub-Saharan Africa have defined virological suppression using a <400 copies/mL threshold, in our study we used a more conservative definition (threshold of <50 copies/mL). In secondary analyses where viral suppression was defined using the <400 copies/mL definition, 86% of patients had suppressed viral load, 299 (88%) in decentralized health facilities and 234 (84%) at central level. These results are similar to those recently reported in South Africa, Zambia, and Cameroon [12], [21], [23], [36]–[38]. The high proportion of patients with viral load measurements between 50 and 400 copies/mL is surprising (29% overall, 69 patients in centralized and 107 in decentralized health facilities) and deserves further investigation. The evaluation of long-term immunovirological patient outcomes in the program could help to understand better the implications of this finding.

In our analysis women and patients who had no history of cART use before enrolment in the HIV program of Chiradzulu were more likely to have gains of CD4 counts ≥100 cells/µL and virological suppression more likely to be observed in patients aged 35 years or more. Higher immune recovery was also reported in previous studies [37], [39]–[42]. In a recent analysis including 4 large HIV programs in sub-Saharan Africa, we found higher immune recovery in women than in men, estimated that sex differences increased by an average of 20 CD4 cells per year in the first 6 years after cART start and also that the time needed to reach 500 cells/µL was shorter for women [42]. As in our analysis, similar gains in CD4 cell counts but higher percentages of patients with viral suppression were also reported in a matched case-control study comparing immunovirological outcomes in older and younger patient (101 patients aged 50–79 years and 202 aged 21–39 years old) in the United States [43].

The burden of HIV infection on health systems and service providers in Malawi is overwhelming. The growing volume of patients receiving cART and the effects on non-HIV related services causes concern, raising questions about the most cost-effective strategies to ensure access to cART while maintaining acceptable treatment outcomes in HIV patients, and the optimal achievable level of task delegation to less-qualified personnel. Scarcity of qualified health care staff, limited access to laboratory testing, unreliable water and electricity supply, and poor road infrastructure are some of the challenges faced when HIV care is provided in rural areas, where the majority of patients in this population live. The decentralization of HIV care and cART provision from central hospitals to rural decentralized health facilities provides an opportunity for improved access. Wide community uptake of cART services and delegation of clinical tasks traditionally performed by physicians to well trained and supervised non-physician health staff have contributed to the achievements of the program.

The findings of this study need to be interpreted in light of some limitations. First, the retrospective evaluation was based on analysis of routine data, and, despite the mechanisms in place to document mortality among patients (e.g. reporting by family and community tracing workers), ascertainment of death was incomplete. Indeed, in a previous study in the same setting, we found that 54% of adults started on cART and lost to follow-up were dead [25]. Although we are likely to have underestimated mortality in this analysis, we also reported program attrition, defined as a combined endpoint of deaths and lost to follow-up and found similar results (e.g. higher attrition rates in centralized than decentralized health facilities). Second, virological monitoring was not routinely performed and immunovirological outcomes were assessed in patients who were randomly selected among those still receiving care in the program after 1 year of therapy. However, the participation rate in the cross-sectional survey was high (88%) and patients included and excluded from the study had similar characteristics at cART start except for the slightly higher proportion of patients with history of cART use and of women, and the higher proportion of patients in clinical stage 3 or 4 among patients not included in the assessment (Table S1). Third, the proportion of patients achieving good immunovirological outcomes might have been overestimated, since poorer outcomes are expected to be found in patients with more advanced HIV disease, which is the case for patients who died or were lost to follow-up during the first months of therapy. Nevertheless, failure rates derived from the sensitivity analysis estimated that only 9% of patients treated in the program would be in virological failure after receiving cART for one year. Fourth, this in an observational study and patients were therefore not randomized to be followed in decentralized or centralized health facilities. Although investigation of risk factors in this large study was adjusted for a number of major confounding factors, including year of cART start, severity of HIV disease and patient adherence of therapy, residual confounding could still be present. Finally, during the initial phase of cART provision in the program, eligibility for therapy start was primarily based on the clinical condition of the patient (e.g. WHO clinical staging) instead of on the results of CD4 count testing. CD4 cell count information was therefore not available for 9.7% of patients included in the analysis. Nevertheless, sensitivity analyses restricted to patients with complete data did not change the conclusions of the analysis.

In conclusion, one-year and two-year HIV program attrition was lower in decentralized than in centralized health facilities of Malawi and no statistically significant differences in one-year immunovirological outcomes were observed between the two health care levels. Longer follow-up is needed to confirm these favorable results. The value of using health care providers with less formal training in rural settings remains an important area for further investigation in rural African settings of high HIV prevalence.

## Supporting Information

Table S1 **Characteristics of patients included and excluded from the cross-sectional study.**
(DOC)
