# Supplementary material for: Treatment Initiation, Program Attrition and Patient Treatment Outcomes Associated with Scale-Up and Decentralization of HIV Care in Rural Malawi
Source: PLoS One. 2012 Oct 15;7(10):e38044. doi: 10.1371/journal.pone.0038044 (PMC3471893; doi:10.1371/journal.pone.0038044)
Supplement: Table S1 — Characteristics of patients included and excluded from the cross-sectional study. (DOC) [file pone.0038044.s001.doc]

**Table S1.** **Characteristics of patients included and excluded from the cross- sectional study**

|  | **Included** | **Excluded** |
| --- | --- | --- |
| **Patients cART initiated,** n | **618** | **12653** |
| **Sex**, n (%) |  |  |
| Men | 232 (37.5) | 4160 (32.9) |
| Women | 386 (62.5) | 8493 (67.1) |
| **Age group**, n (%) |  |  |
| <25 yr | 105 (17.0) | 2399 (19.0) |
| 26-35 yr | 228 (36.9) | 4723 (37.5) |
| 36-42 yr | 133 (21.5) | 2588 (20.5) |
| >43 yr | 152 (24.6) | 2901 (23.0) |
| Unknown | 0 | 42 |
| **Age, years** |  |  |
| Men, median [IQR] | 37.0 [30.3-45.1] | 36.9 [30.3-44.7] |
| Women, median [IQR] | 33.1 [28.1-41.5] | 33.1 [27.1-40.5] |
| **History of cART use**, n (%) | 28 (4.5) | 938 (7.4) |
| **CD4 cell count**, **cells/µL** **^a^** |  |  |
| Tested, n (%) | 550 (89.0) | 8331 (65.8) |
| Median, [IQR] | 174 [104-227] | 163 [95-220] |
| **Clinical stage**, n (%) |  |  |
| 1 or 2 | 306 (50.8) | 4477 (38.8) |
| 3 or 4 | 296 (49.2) | 7057 (61.2) |
| Unknown | 16 | 1119 |

Note: cART, combined antiretroviral therapy; IQR, interquartile range; yr, years.
